# Supplementary material for: Iterative improvement in the automatic modular design of robot swarms
Source: PeerJ Comput Sci. 2020 Dec 7;6:e322. doi: 10.7717/peerj-cs.322 (PMC7924708; doi:10.7717/peerj-cs.322)
Supplement: Supplemental Information 3 [file peerj-cs-06-322-s003.zip › argos3/doc/api/standalone/a00458_source.html]

ARGoS: plugins/robots/foot-bot/simulator/footbot\_distance\_scanner\_default\_actuator.cpp Source File


- Main Page
- Related Pages
- Namespaces
- Classes
- Files

- File List
- File Members

# plugins/robots/foot-bot/simulator/footbot\_distance\_scanner\_default\_actuator.cpp

Go to the documentation of this file.

```
00001 
00007 #include "footbot_distance_scanner_default_actuator.h"
00008 
00009 namespace argos {
00010 
00011          const Real CFootBotDistanceScannerDefaultActuator::RPM_TO_RADIANS_PER_SEC = ARGOS_PI / 30.0f;
00012 
00013    /****************************************/
00014    /****************************************/
00015 
00016    CFootBotDistanceScannerDefaultActuator::CFootBotDistanceScannerDefaultActuator() :
00017       m_fDesiredRotationSpeed(0.0f),
00018       m_unDesiredMode(CFootBotDistanceScannerEquippedEntity::MODE_OFF) {}
00019 
00020    /****************************************/
00021    /****************************************/
00022 
00023    void CFootBotDistanceScannerDefaultActuator::SetRobot(CComposableEntity& c_entity) {
00024       m_pcDistanceScannerEquippedEntity = &(c_entity.GetComponent<CFootBotDistanceScannerEquippedEntity>("distance_scanner"));
00025       m_pcDistanceScannerEquippedEntity->Enable();
00026    }
00027 
00028    /****************************************/
00029    /****************************************/
00030 
00031    void CFootBotDistanceScannerDefaultActuator::SetAngle(const CRadians& c_angle) {
00032       m_unDesiredMode = CFootBotDistanceScannerEquippedEntity::MODE_POSITION_CONTROL;
00033       m_cDesiredRotation = c_angle;
00034    }
00035 
00036    /****************************************/
00037    /****************************************/
00038 
00039    void CFootBotDistanceScannerDefaultActuator::SetRPM(Real f_rpm) {
00040       m_unDesiredMode = CFootBotDistanceScannerEquippedEntity::MODE_SPEED_CONTROL;
00041       m_fDesiredRotationSpeed = f_rpm * RPM_TO_RADIANS_PER_SEC;
00042    }
00043 
00044    /****************************************/
00045    /****************************************/
00046 
00047    void CFootBotDistanceScannerDefaultActuator::Enable() {
00048       m_unDesiredMode = CFootBotDistanceScannerEquippedEntity::MODE_POSITION_CONTROL;
00049    }
00050 
00051    /****************************************/
00052    /****************************************/
00053 
00054    void CFootBotDistanceScannerDefaultActuator::Disable() {
00055       m_unDesiredMode = CFootBotDistanceScannerEquippedEntity::MODE_OFF;
00056    }
00057 
00058    /****************************************/
00059    /****************************************/
00060 
00061    void CFootBotDistanceScannerDefaultActuator::Update() {
00062       m_pcDistanceScannerEquippedEntity->SetMode(m_unDesiredMode);
00063       if(m_unDesiredMode == CFootBotDistanceScannerEquippedEntity::MODE_POSITION_CONTROL) {
00064          m_pcDistanceScannerEquippedEntity->SetRotation(m_cDesiredRotation);
00065       }
00066       else if(m_unDesiredMode == CFootBotDistanceScannerEquippedEntity::MODE_SPEED_CONTROL) {
00067          m_pcDistanceScannerEquippedEntity->SetRotationSpeed(m_fDesiredRotationSpeed);
00068       }
00069    }
00070 
00071    /****************************************/
00072    /****************************************/
00073 
00074    void CFootBotDistanceScannerDefaultActuator::Reset() {
00075       m_cDesiredRotation = CRadians::ZERO;
00076       m_fDesiredRotationSpeed = 0.0f;
00077       m_unDesiredMode = CFootBotDistanceScannerEquippedEntity::MODE_OFF;
00078    }
00079 
00080    /****************************************/
00081    /****************************************/
00082 
00083    REGISTER_ACTUATOR(CFootBotDistanceScannerDefaultActuator,
00084                      "footbot_distance_scanner", "default",
00085                      "Carlo Pinciroli [ilpincy@gmail.com]",
00086                      "1.0",
00087                      "The foot-bot distance scanner actuator.",
00088                      "This actuator controls the foot-bot distance scanner. For a complete\n"
00089                      "description of its usage, refer to the ci_footbot_distance_scanner_actuator\n"
00090                      "file.\n\n"
00091                      "REQUIRED XML CONFIGURATION\n\n"
00092                      "  <controllers>\n"
00093                      "    ...\n"
00094                      "    <my_controller ...>\n"
00095                      "      ...\n"
00096                      "      <actuators>\n"
00097                      "        ...\n"
00098                      "        <footbot_distance_scanner implementation=\"default\" />\n"
00099                      "        ...\n"
00100                      "      </actuators>\n"
00101                      "      ...\n"
00102                      "    </my_controller>\n"
00103                      "    ...\n"
00104                      "  </controllers>\n\n"
00105                      "OPTIONAL XML CONFIGURATION\n\n"
00106                      "None for the time being.\n",
00107                      "Usable"
00108       );
00109 
00110 }
```

---

Generated on 10 Jul 2018 for ARGoS by 
 1.6.1
